# Physiological Effects of the Iodide of Potassium

**Published:** 1844-09

**Authors:** 


*Physiological Effects of the Iodide of Potassium.*—M. Ricord, who has had such large experience in the use of the iodide of potassium in different doses, has made valuable observations on its physiological action. The following may be regarded as a summary of these.

The *skin* is very readily affected by the iodide. as is shown in the frequent eruptions on the face and shoulders, and even other parts of the body, of those who have been subjected for some time to the influence of this medicine.

The *digestive functions* under the use of the iodide of potassium are generally quickened in their functions in a favorable manner; the appetite is increased, and a general fulness of frame is the frequent consequence. Under certain circumstances, however, this medicine may give rise to pathological effects on the digestive passages. One of these, and the most remarkable and at the same time common, consists in a pain seated in the great curvature of the stomach, and which, as it is described by the patients, might be taken for a pleurodynic pain of the left hypochondrium. This pain is sometimes quite sharp, but without being accompanied by any augmentation of thirst or derangement of the appetite, or appearance of the tongue indicating any gastric derangement, or, finally, without there being any reactive excitement of the circulation.

*Salivation.*—In a great number of subjects, the iodide of potassium gives rise to a copious salivation, resembling that of pregnant women. The buccal mucous membrane is not inflamed. the salivary glands are not swelled, and the breath has not the fetor characteristic of mercurial ptyalism.

The *quantity of urine* is almost always increased, and often in a very remarkable manner, under the influence of the iodide of potassium.

The *circulation* has not appeared to M. Ricord to be sensibly affected by the iodide, but in some instances he thought that it rendered the blood less plastic, and gave rise to a predisposition to nasal and pulmonary, and sometimes to intestinal, hemorrhages. Sometimes it has given rise to a kind of ophthalmia, to which he proposes the name of edematous catarrh.

Under the influence of the iodide of potassium there often supervenes, as in simple coryza, obstruction in the nasal fossæ, sniffling, seldom sneezing; the mucous secretion is considerably increased; but the flux has this peculiarity, that it is commonly less viscous, and displays no tendency to pass into the purulent stage. Sometimes this variety of coryza is very annoying. Twice during the year (1843), the editor of the *Annuaire Therapeutique*, &c., whose description we are now following, says that it has been, within his own observation, accompanied by quite a severe cephalalgia, so as to cause alarm for the result; and yet the patients had not been taken more than fifteen grains of the iodide.

Sometimes the iodic coryza is succeeded by a peculiar kind of bronchitis, with cough and labored respiration.

The action of iodide of potassium on the *nervous system*, though of unfrequent occurrence, is quite remarkable. M. Ricord has seen to supervene, in some subjects, a little cerebral excitement and symptoms of a slight congestion, which have given rise to something analogous to the intoxication produced by alcoholic liquors. Occasionally spasmodic movements and slight *subsultus tendinum* are seen.—*Bulletin Med. Sciences.*

